# *Aronia melanocarpa* Extract Fermented by *Lactobacillus plantarum* EJ2014 Modulates Immune Response in Mice

**DOI:** 10.3390/antiox10081276

**Published:** 2021-08-11

**Authors:** Md. Sekendar Ali, Eon-Bee Lee, Seung-Jin Lee, Sam-Pin Lee, Naila Boby, Kyoungho Suk, Biruk Tesfaye Birhanu, Seung-Chun Park

**Affiliations:** 1 Department of Biomedical Science and Department of Pharmacology, School of Medicine, Brain Science and Engineering Institute, Kyungpook National University, Daegu 41944, Korea; alipharm2000@gmail.com (M.S.A.); ksuk@knu.ac.kr (K.S.); 2 Laboratory of Veterinary Pharmacokinetics and Pharmacodynamics, College of Veterinary Medicine, Kyungpook National University, Daegu 41566, Korea; eonbee@gmail.com (E.-B.L.); nailaboby@knu.ac.kr (N.B.); 3 Department of Pharmacy, International Islamic University Chittagong, Kumira, Chittagong 4318, Bangladesh; 4 Development and Reproductive Toxicology Research Group, Korea Institute of Toxicology, Daejeon 34114, Korea; dvmleesj@naver.com; 5 Department of Food Science and Technology, Keimyung University, Daegu 42601, Korea; splee@knu.ac.kr

**Keywords:** *Aronia melanocarpa*, *Lactobacillus plantarum*, fermentation, GABA, polyphenols, RAW 264.7 cells, proinflammatory cytokines, immunomodulation

## Abstract

The present study aimed to assess the immunomodulatory effects of fermented *Aronia melanocarpa* extract (FAME) on RAW 264.7 cells and BALB/c mice. *Aronia melanocarpa* fruit was fermented with *Lactobacillus plantarum* EJ2014 by adding yeast extract and monosodium glutamate for 9 days at 30 °C to produce γ-aminobutyric acid (GABA). After fermentation, significant GABA production was noted, along with minerals, polyphenols, and flavonoids (*p* < 0.05). The polyphenol content was confirmed by liquid chromatography with tandem mass spectrometry (LC–MS/MS) analysis. RAW 264.7 cells were stimulated with lipopolysaccharide (LPS, 1 μg/mL) in the presence or absence of FAME, and proinflammatory cytokine contents were measured by qPCR. In the in vivo experiment, female BALB/c mice were administered 125, 250, and 500 mg/kg of FAME for 21 days. FAME treatment increased neutrophil migration and phagocytosis (*p* < 0.05). It also increased splenocyte proliferation, CD4^+^ and CD8^+^ T-cell expression, and lymphocyte proliferation. Furthermore, it increased IFN-γ, IL-2, and IL-4 cytokine levels in a dose-dependent manner (*p* < 0.05). However, it decreased TNF-α and IL-6 levels (*p* < 0.05). These results indicate that FAME fortified with GABA including bioactive compounds exerts anti-inflammatory effects by inhibiting proinflammatory cytokines in RAW 264.7 cells and modulates immune response in mice. Thus, FAME could be a potential therapeutic agent for inflammatory disorders.

## 1. Introduction

Medicinal plants play an important role in the treatment of diseases because they are rich sources of different phytochemicals [1]. The use of different plants as complementary drugs is gaining attention worldwide because of their low cost and high therapeutic potential. *Aronia melanocarpa* (family: Rosaceae), also known as *Photinia melanocarpa*, is native to the USA and Canada. The fruits of *A. melanocarpa* (chokeberries) are small, dark, and cherry-like. They have many reported health benefits and can be used to treat heart disease, high cholesterol, and hypertension, among others [2,3]. These fruits contain great amounts of polyphenols, flavonoids, and anthocyanins and have antioxidant, anti-inflammatory, and anticancer properties, thereby exerting several health benefits [2,4,5]. Worldwide, it is a well-known fact that foods fermented by lactic acid bacteria have an immune-enhancing effect. Therefore, many recent studies have assessed the immune activity of medicinal plants following lactic acid bacterial fermentation [6,7]. When medicinal plants are fermented by lactic acid bacteria, bioconversion occurs. At the same time, immunity is enhanced through the production of beneficial bioactive metabolites, such as essential amino acids and fatty acids, vitamins, and polyphenolic compounds, thereby making fermented foods more functional than nonfermented ones [8]. Studies have revealed that lactic acid bacteria can synthesize γ-aminobutyric acid (GABA) in various food media [9,10,11,12]. A recent study demonstrated that *Lactobacillus brevis* CRL 2013 can efficiently convert monosodium glutamate (MSG) to GABA, corroborating that *L. plantarum* EJ2014 (used in the present study) could also be used to produce GABA [13], a ubiquitous nonprotein amino acid possessing diverse physiological activities and many potential health benefits [14]. The use of artificially produced GABA is associated with legal issues in several countries [15,16]. Therefore, GABA produced using *L. plantarum* EJ2014 as a starter culture during the fermentation of aronia juice could be an alternative functional food. The strain *L. plantarum* EJ2014 used in the present study is a representative probiotic that has long been used for producing fermented products. It has been reported to play a role in intestinal regulation and immune enhancement [17]. These beneficial effects have led to the development of functional food products based on *Lactobacillus* strains [18]. 

Previous studies almost exclusively focused on the immunomodulatory activities of nonfermented *A. melanocarpa* extract. Therefore, in the present study, we assessed the anti-inflammatory effects of fermented aronia extract (FAME) on RAW 264.7 cells. Moreover, we confirmed whether the administration of FAME could modulate innate and adaptive immunity by producing compounds such as GABA, flavonoids, polyphenols, and minerals and increasing or decreasing the neutrophil count, phagocytosis, splenic lymphocyte proliferation, CD4^+^ and CD8^+^ T cell phenotyping, and cytokine expression in BALB/c mice.

## 2. Materials and Methods

### 2.1. Aronia Fruit and Reagents

Aronia fruit was purchased at the local market (Sangju, Gyeongsangbuk-do, Korea). Yeast extract and MSG were procured from Choheung Co. (Ansan, Korea) and Yakuri Pure Chemicals Co. Ltd. (Kyoto, Japan), respectively. High-quality grade reagents were used in all the experiments. The chemicals and media used in the present study were purchased from Sigma-Aldrich (Sigma-Aldrich, Yongin, Korea) and Difco (Becton, Dickinson and Company, Franklin Lakes, NJ, USA), respectively, unless stated otherwise.

### 2.2. Fermentation of FAME

For lactic acid fermentation, *L. plantarum* EJ2014 (KCCM 11545P) was isolated from rice bran, and molecular analysis was performed using PCR at the Food Processing Resource Development Laboratory of Keimyung University, Korea. Then, *L. plantarum* EJ2014 was cultured on a *Lactobacillus* MRS broth (Difco, BD, USA) at 30 °C for 24 h and used for fermentation of *A. melanocarpa* to produce GABA and other bioactive compounds. Briefly, aronia fruits without the stem were ground using a cutter miller (Philips, Amsterdam, The Netherlands) to prepare juice. The fruit juice (10%) was mixed with 1% yeast extract, 1% skim milk, and 2% MSG followed by autoclave for 15 min at 121 °C. Then, *L. plantarum* EJ2014 starter culture was inoculated and incubated at 30 °C for 9 days for fermentation. The final fermented product of *A. melanocarpa* was lyophilized with a vacuum freeze dryer (Operon Advantech Co., Ltd., Gyeonggi, Korea) and stored at −20 °C until use. The fermented mixture was analyzed for GABA production using thin-layer chromatography (TLC) [19] and compared with a nonfermented control sample. Before and during the fermentation, samples were collected for the measurement of different parameters, such as the pH, reducing sugar content, polyphenol content, flavonoid content, anthocyanin content, and viable cell count. Moreover, the α-α-diphenyl-β-picrylhydrazyl (DPPH) radical scavenging activity, mineral content, and phosphorus content were assessed before and at the end of the fermentation.

### 2.3. Analysis of FAME

#### 2.3.1. GABA Content 

For the qualitative analysis of GABA, silica gel TLC plates (10 × 20 cm) and a square chamber (30 × 25 × 10 cm) for solvent development were used. The developed solvent consisted of n-butyl alcohol, glacial acetic acid, and distilled water at a ratio of 6:2:2 (*v/v/v*) and was saturated at room temperature for at least 4 h. The fermented sample (2 μL) and standard solution (1% GABA) were spotted on the TLC plate for development and dried at room temperature. As a coloring reagent, 0.2% ninhydrin solution was sprayed on the dried TLC plate, which was then developed for about 5 min at 100 °C to confirm the presence of GABA spots of the fermented sample. Furthermore, free amino acid (glutamic acid) and GABA were analyzed by high-performance liquid chromatography (HPLC). The dried sample was derivatized with 20 μL of a solution containing phenyl isothiocyanate (PITC), methanol (MeOH), triethylamine (TEA), and water (H_2_O) (MeOH:H_2_O:TEA:PITC = 7:1:1:1); reacted at room temperature for 30 min; and centrifuged for 5 min at 14,000 rpm. The supernatant was filtered with a 0.45 μL syringe filter and analyzed using a C_18_ column (Waters Nova-Pak 4 μm) (Waters Corporation, Milford, MA, USA) on an HPLC device (Hewlett Packard 1100 series, Palo Alto, CA, USA) at an absorbance of 254 nm. The mobile solvent consisted of 140 mM NaHAc, 0.15% TEA, 6% CH_3_CN, and 0.015% EDTA. 

#### 2.3.2. Antioxidant Activity 

The DPPH free radical scavenging activity of FAME fermented by *L. plantarum* EJ2014 was measured using a previously described method [20]. The sample (160 μL) was added to 40 μL of 0.15 mM DPPH solution in ethanol, and the absorbance was measured at 517 nm. The activity of the sample was expressed as IC_50_ using ascorbic acid as a positive control.

#### 2.3.3. Mineral Content

The mineral analysis was done for samples collected immediately before fermentation and after fermentation with *L. plantarum* EJ2014 on the 9th day of fermentation. The collected samples were immediately sent to The Center for Traditional Microorganism Resources, Keimyung University (Food Inspection Testing Lab, Livestock Inspection Testing Lab)**.** The mineral content in FAME was determined by heating 10 g of the extract at 550–600 °C for 12 h. The residue was solubilized in 10 mL of HCl and then filtered. The sample was analyzed using an inductively coupled plasma optical emission spectrometer (ICP-OES; Optima 700DV, Perkin Elmer, Waltham, MA, USA), and a standard curve was used to measure the concentrations. The mineral standard references were purchased from AccuStandard Inc. (New Haven, CT, USA) and Sigma-Aldrich Co. (St. Louis, MO, USA). The solvent used for the extraction was of normal grade, and the analysis was performed using HPLC-grade solvents (Sigma-Aldrich Co, St. Louis, MO, USA). The analysis value reliability was confirmed using a quality control chart for internal analysis quality control of inorganic substances (Quality Control chart, QC chart). For analyzing minerals (K, Ca, Mg, Na, Fe, P), standard reference material (SRM) 1869 was used to determine the analytical parameters of the method such as limit of detection (LOD), limit of quantification (LOQ), sensitivity, accuracy, and precision (Supplementary Table S1).

#### 2.3.4. pH, Acidity, and Viable Cell Count 

The pH of the extract was measured using a pH meter (model 420+ Thermo, Washington, DC, USA). The titratable acidity was determined by adding 1 mL of the extract to 9 mL of distilled water and calculating the percentage of lactic acid (*v/v*) consumed by 0.1 N NaOH at pH 8.3. The number of viable bacterial cells was counted and expressed as CFU/mL by plating the sample on an MRS agar plate after serial dilution and incubation for 24 h at 30 °C.

#### 2.3.5. Polyphenol and Flavonoid Content

The polyphenol content was measured using the Folin–Ciocalteu method [21]. Fermentation broth containing the extract was diluted 2, 5, and 10 times, and the initial absorbance was measured at 700 nm by taking 60 μL of each sample. Then, 60 μL of 2-fold-diluted Folin reagent was added before the mixture was homogenized and allowed to stand for 3 min. Following this, 60 μL of 10% Na_2_CO_3_ was added and reacted in a constant temperature incubator at 30 °C for 60 min before reading the absorbance at 700 nm. The total polyphenol content was measured from a standard curve using gallic acid as a reference material. The flavonoid content was measured as described by Nieva Moreno et al. [22]. In brief, the sample (125 μL) was added to 1080 μL of 80% ethanol, 30 μL of 10% aluminum nitrate, and 30 μL of 1 M potassium acetate. The mixture was incubated at room temperature for 40 min, and the absorbance was measured at 415 nm. The total flavonoid content was measured from a standard curve using quercetin as a standard.

#### 2.3.6. Anthocyanin Content

The anthocyanin pigment content plays an important role in determining food color and quality. The anthocyanin content in FAME was determined using a previously described method [23]. In brief, anthocyanin stock solution (1 mg/L) was prepared with 5% *v/v* perchloric acid solution to generate a standard curve. The test solution was prepared at different concentrations (100–500 μg/mL), and 5% *v/v* perchloric acid was added, followed by stirring for 30 min. The absorbance was measured at 450 nm, and the anthocyanin content was measured by referring to the standard calibration curve.

#### 2.3.7. Reducing Sugar Content

The reducing sugar content was measured using the dinitrosalicylic acid (DNS) method [24]. The sample (5 mL) was centrifuged at 13,200 rpm for 10 min, and 1 mL of the diluted supernatant was mixed with 3 mL of the DNS reagent and kept at 100 °C for 5 min. The absorbance was measured at 550 nm using a refractometer (Pocket Refractometer Pal-3, Atago, Japan) after cooling at room temperature in the dark for 40 min. The reducing sugar content was calculated from a standard curve using glucose as a standard and was based on three readings.

#### 2.3.8. LC–MS/MS Analysis 

LC–MS/MS analysis was performed using an Accela UHPLC system (Thermo Fisher Scientific, CA, USA) coupled with a high-resolution LTQ-Orbitrap XL hybrid mass spectrometer (Thermo Electron, Bremen, Germany) via an ESI interface. Sample separation was performed at 40 °C using a Waters BEH C18 column (2.1 × 150 mm, 1.7 μm). Water (A) and acetonitrile (B) were used as the mobile phase, with 0.1% formic acid being added to both solvents. The flow rate was adjusted to 400 μL/min. The elution gradient was as follows: 0% B (0–1 min), 0–40% B (1–20 min), 40–100% B (20–24 min), and 100% B (24–27 min). The sample (2 μL) was injected, and the analysis was performed using PDA at 200–600 nm and MS at *m/z* 100–1000 in the positive ion mode. The conditions of the ESI source were similar to those of a previous study. The compounds were identified using the reference literature [25], high-resolution mass spectra, and MS/MS spectral library search [26].

### 2.4. RAW 264.7 Cells and In Vitro Study Design

**Figure 1:**
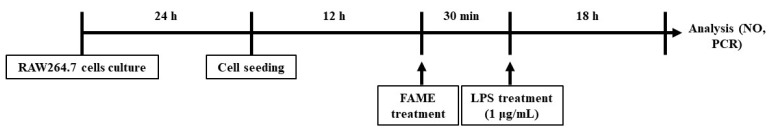
In vitro experimental scheme.

RAW 264.7 cells were used to conduct an in vitro experiment for assessing nitric oxide (NO) production and proinflammatory cytokine mRNA. The experimental timeline is shown in Figure 1.

#### 2.4.1. NO Assay

RAW 264.7 cells (Korean Cell Line Bank, Seoul, Korea) were cultured for 24 h to reach 80% confluence. Following this, 1 × 10^5^ cells/well were allowed to adhere for 12 h in a 24-well plate. After 30 min of treatment with different extract concentrations (0.025, 0.05, 0.1, and 0.2 mg/mL), the cells were incubated with lipopolysaccharide (LPS) (1 µg/mL) for 18 h. The production of NO in the cell culture medium was measured as the (NO_2_) level at 540 nm and quantified from a standard curve generated using sodium nitrite (NaNO_2_). The cytotoxicity of the extracts in RAW 264.7 cells was determined using the 3-(4,5-dimethylthiazol-2-yl)-2,5-diphenyl tetrazolium bromide (MTT) assay, and the absorbance was measured at 570 nm [27].

#### 2.4.2. Total RNA Extraction and Traditional and Quantitative Real Time RT-PCR

Total RNA was extracted from the cells using TRIzol reagent (Life Technologies, Carlsbad, CA, USA), and 5 μg of RNA from every sample was reverse-transcribed into cDNA using the AccuPower RT premix (Bioneer, Daejon, Korea). Reverse transcription PCR was performed using the GeneAmp PCR System 9700 (Applied Biosystems, Waltham, MA, USA). To analyze the PCR products, 6 μL of each PCR reaction mixture was electrophoresed on 1.5% agarose gel and detected under ultraviolet (UV) light. Relative mRNA expression was determined using the CFX96 Touch PCR Detection System (Bio-Rad, Singapore). PCR amplification was performed using the following primers, with β-actin being used as a standard control: IL-1β (Genebank accession No.: NM_008361.4) forward (F): TGAGCACCTTCTTTTCCTTCA and reverse (R): TTGTCTAATGGGAACGTCACAC; IL-6 (Genebank accession No.: NM_031168.2) F: TAATTCATATCTTCAACCAAGAGG and R: TGGTCCTTAGCCACTCCTTC; TNF-α (Genebank accession No.: LN874395.1) F: CTGTAGCCCACGTCGTAGC and R: GGTTGTCTTTGAGATCCATGC; iNOS (Genebank accession No.: NM_001313922.1) F: TGTGGCTACCACATTGAAGAA and R: TCATGATAACGTTTCTGGCTCTT; Cox-2 (Genebank accession No.: NM_011198.4) F: CACTACATCCTGACCCACTT and R: ATGCTCCTGCTTGAGTATGT; TLR4 (Genebank accession No.: NM_021297.3) F: GACCTCAGCTTCAATGGTGC and R: TATCAGAAATGCTACAGTGGATACC; and β-actin (Genebank accession No.: NM_007393.5) F: GTCATCACTATTGGCAACGAG and R: TTGGCATAGAGGTCTTTACGG. Quantitative real-time PCR was performed under the following cyclic conditions: enzyme activation and initial denaturation for 5 min at 95 °C and 35 cycles of amplification at 95 °C for 10 s, followed by 56 °C (TNF-α, IL-6, Cox-2, and TLR4) or 60 °C (iNOS and IL-1β) for 20 s. 

### 2.5. Animals and Study Design

All experimental protocols involving animals were approved by the Kyungpook National University Ethical Committee (KNU-2017-50). Five-week-old female BALB/c mice were procured (ORIENT BIO, Republic of Korea) and acclimated to laboratory conditions for 7 days. Female mice were used due to their strong immune response [28,29]. The animal room was maintained at a relative humidity of 50–65% and a temperature of 20–24 °C with 12 h light and dark periods. The mice were fed a standard pellet diet and provided ad libitum filtered tap water access. After adaptation to the laboratory conditions, the mice were randomly divided into six groups of eight each (Table 1).

**Figure 2:**
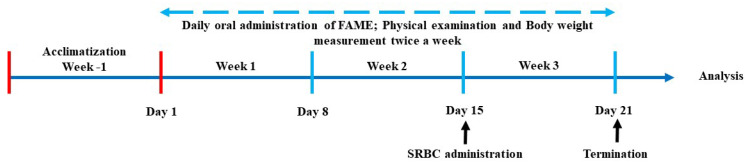
Outline of the in vivo experiment.

The mice in the treatment groups received a daily oral dose of 200 µL FAME at 125, 250, or 500 mg/kg for 21 days (Figure 2). On day 15, the mice were immunized by the intraperitoneal administration of 5.0 × 10^8^/mL sheep red blood cells (SRBCs). Their body weight was measured regularly to adjust dosing volumes. After 21 days, the mice were sacrificed and their blood and organs were collected. Blood was collected in heparinized tubes and centrifuged at 10,000 rpm for 5 min. Plasma was separated and stored at −70 °C until use in enzyme-linked immunosorbent assay (ELISA), and organs were weighed and processed accordingly.

#### 2.5.1. Acute Oral Toxicity Test

To assess the potential oral toxicity of FAME, 5-week-old female Sprague–Dawley rats were used. The rats were divided into two groups containing five rats each: normal and treated. Each rat was orally administered a single dose of FAME (2000 mg/kg body weight) according to OECD guidelines 423 [30]. A standard pellet diet and distilled water were supplied ad libitum throughout the observation period. The rats were constantly monitored for abnormal physical signs and clinical symptom changes for 14 days after the administration of FAME. The food and water intake and the body weight changes were measured twice a week. Finally, the rats were sacrificed after euthanasia. Following this, their major organs were collected, inspected for abnormal gross lesions, and compared between the control and treated groups. 

#### 2.5.2. Neutrophil Isolation and Counting

Neutrophils were isolated from whole blood using Histopaque 1077 (Sigma-Aldrich, St. Louis, MO, USA). In brief, equal volumes of whole blood and Histopaque solution were mixed and centrifuged at 400× *g* for 30 min at room temperature. The opaque interface was collected after carefully removing the upper layer. The interface was washed thrice by adding isotonic phosphate-buffered saline (PBS) and centrifuged at 250× *g* for 10 min. Finally, Dulbecco’s Modified Eagle Medium (DMEM, Sigma-Aldrich, St. Louis, MO, USA) supplemented with 10% fetal bovine serum (FBS) was added to the pellet, and the cells were counted using trypan blue dye.

#### 2.5.3. Neutrophil Migration Assay

The neutrophil migration assay was conducted using the Cytoselect 24-Well Cell Migration Assay Kit (3 µm, fluorometric format) (Cell Biolabs, Inc., San Diego, CA, USA), according to the manufacturer’s protocol. In brief, neutrophils were isolated as mentioned above, followed by the addition of 300 µL of 5 × 10^6^ cells/mL in serum-free DMEM to the inside of the insert and 500 µL of medium supplemented with 10% FBS to the lower part of the migration plate. After overnight incubation, the medium from the inside of the insert was aspirated before the insert was transferred to a clean well containing 200 µL of cell detachment solution and incubated at 37 °C for 30 min. The cells were completely dislodged from the underside of the membrane, and an additional 400 µL of medium from the migratory cells was transferred to the detachment solution. Following this, 180 µL of the mixture was transferred to a 96-well plate, and 60 µL of 4× Lysis Buffer/CyQuant GR dye (Thermo Fisher Scientific, Waltham, MA, USA) was added before incubating for 20 min at room temperature. Finally, 200 µL of the mixture was transferred to a new 96-well plate. The fluorescence was measured at 480 nm using a fluorescence plate reader and expressed as the relative fluorescence unit (RFU).

#### 2.5.4. Neutrophil Phagocytic Activity

The phagocytic activity of neutrophils was analyzed using the Cytoselect 96-Well Phagocytosis Assay Kit with *Escherichia coli* substrate (Cell Biolabs, Inc., Waltham, CA, USA). Neutrophils were collected as described above, followed by the addition of 100 µL of 10^6^ cells/mL into a 96-well plate before adding 10 µL of *E. coli* suspension. The mixture was incubated for 6 h at 37 °C under 5% CO_2_. The medium was changed after centrifugation, and the cells were washed with 200 µL of cold PBS. Then, the cells were incubated for 5 min with 100 µL of fixation solution at room temperature. After removing the fixation solution, the cells were washed with PBS before 100 µL of blocking solution was added and incubated for 30 min at room temperature on an orbital shaker. Finally, the reaction was initiated by adding 100 µL of the substrate, followed by incubation for 20 min before adding 100 µL of stop solution. The absorbance was measured at 450 nm using a Versamax plate reader (Molecular Devices, San Jose, CA, USA). 

#### 2.5.5. Preparation of Splenocytes

Splenocytes were prepared as described by Ahmad et al. [31]. In brief, after sacrificing the mice, the spleen was removed aseptically. The collected spleen tissue was placed into a sterile tube with Hank’s balanced salt solution (HBSS) (Gibco Life Technologies, New York, NY, USA) and kept on ice. The spleen tissue was then minced, pressed through a 70 μm fine nylon cell strainer (BD Biosciences, CA, USA) using a plunger at the end of a 3 mL syringe, and washed with an excess amount of HBSS. The obtained cell suspensions were centrifuged for 5 min at 1600 rpm. After discarding the supernatant, the collected pellet was resuspended in 1 mL prewarmed red blood cell (RBC) lysis buffer solution (Sigma, Welwyn Garden City, UK). The cells were incubated for 1 min at 37 °C to lyse RBCs, followed by the addition of HBSS. Again, the cells were centrifuged at 1600 rpm at 4 °C for 5 min. The cell pellets were washed thrice with PBS. Next, the supernatant was removed and the pellets were resuspended in RPMI-1640 medium (Sigma-Aldrich, St. Louis, MO, USA) supplemented with 10% FBS and 1% penicillin–streptomycin (P/S) solution. Finally, the trypan blue dye exclusion method was used to count the cells with a hemocytometer.

#### 2.5.6. Splenic Lymphocyte Proliferation Assay

In total, 4 × 10^6^ cells/mL splenocytes from the treatment and control groups were seeded (200 μL/well) into a 96-well plate in RPMI-1640 medium containing 10% FBS and 1% P/S. Splenocytes from each group were cultured in the presence of the mitogen concanavalin A (Con A, 5 μg/mL) or LPS (10 μg/mL) and in the absence of any of the mitogens for 72 h at 37 °C. After adding 5 mg/mL of MTT reagent solution, the absorbance was measured at 570 nm. The proliferation index was determined by dividing the optical density (OD) of the sample by the control OD value.

$$\mathrm{Proliferation}\;\mathrm{index}\;=\frac{\mathrm{OD}\;\mathrm{of}\;\mathrm{treated}\;\mathrm{cells}\;}{\mathrm{OD}\;\mathrm{of}\;\mathrm{control}\;\mathrm{cells}}$$

#### 2.5.7. Flow Cytometric Analysis for T-Lymphocyte Phenotyping

For T-lymphocyte phenotyping, 10^6^ cells/100 μL splenocytes were harvested into fluorescence-activated cell sorting (FACS) tubes. Cell blocking was accomplished by adding IgG blocking solution (1 μg IgG/10^6^ cells) and incubating at room temperature for 15 min. In total, 10 μg/mL conjugating antibodies were added and vortexed, followed by the incubation of cells for 30 min in a dark place. Unbound antibodies were removed by washing with flow cytometry staining buffer, and suspended cells were centrifuged for 5 min at 300× *g*. For flow cytometric analysis, the pelleted cells were resuspended in 200 μL of FACS buffer solution. Finally, data acquisition and analysis were performed using multicolor FACS with FACSDiva version 6.1.3 software on BD FACSAria III (BD Biosciences, San Diego, CA, USA). The percentages of different T-lymphocyte subpopulations (CD4^+^ and CD8^+^) were calculated to compare the expression levels among different groups.

#### 2.5.8. Determination of Cytokine Concentrations

The experiment was performed according to the manufacturer’s protocol (Elabscience). In brief, 50 μL of microparticle cocktail was added to the well of a microplate together with an equal volume of the sample, standard, or control and incubated for 2 h at room temperature on an orbital microplate shaker. After washing four times with diluted wash buffer, 50 μL of diluted biotin antibody cocktail was added and incubated for 1.5 h at room temperature on the shaker. Again, after washing four times with wash buffer, 50 μL of diluted streptavidin–PE was added to each well and incubated for 30 min at room temperature on the shaker. Following this, 100 μL of wash buffer was added to resuspend the microparticles and incubated for 2 min. The absorbance was then measured at 450 nm using a Luminex MAGPIX analyzer (Luminex, Austin, TX, USA). The individual analyte in the sample reacted with the corresponding antibody, which was attached to a specific number of beads. The cytokine concentration in the sample was measured by generating a standard curve using five-parameter logistic curve fitting (Masterplex QT 2010, MiraiBio, Hitachi, CA, USA) and multiplied by the dilution factor.

### 2.6. Statistical Analysis

The obtained data were analyzed using one-way and two-way analyses of variance, followed by Dunnett’s test and Tukey’s test using GraphPad Prism 7 software (GraphPad Software Inc., San Diego, CA, USA). The results are expressed as the mean ± standard error of the mean (SEM). *p* values < 0.05 were considered statistically significant.

## 3. Results

### 3.1. GABA Content in FAME

**Figure 3:**
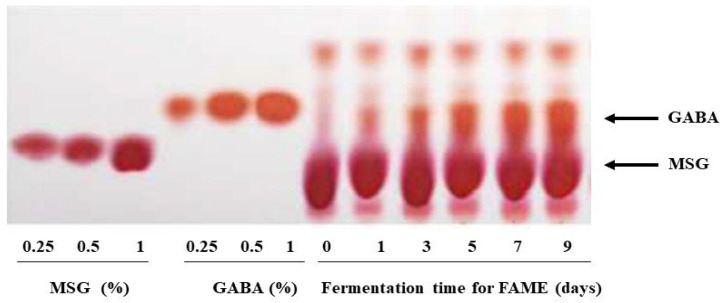
Fermentation effects on GABA production pattern in FAME. Thin-layer chromatogram (TLC) for MSG standard (0.25–1%), GABA standard (0.25–1%), and 5-fold dilution of FAME samples collected at different time intervals (0, 1, 3, 5, 7, and 9 days) during fermentation.

GABA content of FAME was qualitatively determined using TLC (Figure 3). As a control, *L. plantarum* EJ2014 was grown in the same juice without any extra supplementation. GABA production was not detected in the control sample. TLC analysis revealed only a residual amount of MSG but no GABA production before fermentation. However, the GABA concentration gradually increased until day 9 during fermentation (Patent No. 1020170073239). Quantitative analysis (Table 2) revealed no GABA production before fermentation; however, on day 9 of fermentation, significant GABA production was noted (10.4 mg/mL) (*p* < 0.001).

### 3.2. Antioxidant Activity of FAME

The antioxidant activity of FAME was measured by the DPPH radical scavenging activity assay. The results revealed that the IC_50_ value of DPPH decreased from 2.18 to 1.09 mg/mL after fermentation, indicating that the antioxidant activity increased 2-fold as a result of fermentation (Table 2) (*p* < 0.001). This may be attributed to increased contents of anthocyanins, flavonoids, and phenolic compounds, including quercetin.

### 3.3. Mineral Content in FAME 

The mineral composition of FAME was analyzed before and after *L. plantarum* EJ2014 fermentation (Table 2). Compared with nonfermented aronia extract, the potassium, calcium, magnesium, sodium, iron, and phosphorus contents increased by 2.7%, 39.7%, 16.7%, 77%, 11%, and 85.2%, respectively, in FAME (*p* < 0.05). Thus, fermentation by *L. plantarum* EJ2014 increased the mineral content in the extract. 

### 3.4. Changes in pH, Acidity, and Viable Cell Count of FAME

Physicochemical properties (pH and acidity) are important indices for determining the progress of fermentation [32]. Hence, we measured the pH and acidity of the extract before, during, and after culture with yeast extract and MSG at 30 °C for 9 days. The pH was 4.41 before fermentation. It kept decreasing during culture and reached a minimum of 3.31 on day 3. Accordingly, the acidity of FAME increased from 0.74 to the peak value of 1.08 on day 3. However, further fermentation slightly increased the pH to 4.01 on day 9, with a corresponding decrease in acidity (Table 3). The overall growth of *L. plantarum* EJ2014 increased during fermentation (Table 3) (*p* < 0.05). The early viable cell count of the *Lactobacillus* starter was 4.86 × 10^6^ CFU/mL; it increased to 1.25 × 10^9^ CFU/mL on day 1. The viable cell count slightly decreased to 10^8^ CFU/mL on day 3 and continued to decrease to 1.55 × 10^8^ CFU/mL until day 5. Then, the viable cell count was maintained at a steady level of 10⁷ CFU/mL until the end.

### 3.5. Polyphenol and Flavonoid Content in FAME

The effect of *L. plantarum* EJ2014 fermentation on polyphenol and flavonoid production is presented in Table 3. The polyphenol (46.19%) and flavonoid (11.4%) content in FAME increased from day 0 to day 9 during fermentation (*p* < 0.05). The presence of the polyphenol content was further confirmed by LC–MS/MS analysis (Table 4).

### 3.6. Anthocyanin Content in FAME

Anthocyanins are major polyphenolic compounds that can be produced as a result of fermentation. The impact of fermentation on anthocyanin production is presented in Table 3. The anthocyanin content in FAME increased (13.71%) from day 0 to day 9 as a result of fermentation (*p* < 0.05).

### 3.7. Reducing Sugar Content in FAME

The measured reducing sugar contents are shown in Table 3. After 9 days of fermentation, the reducing sugar content in FAME decreased from 4.1 to 0.86% (*p* < 0.05). 

### 3.8. LC–MS/MS Analysis of FAME

As shown in Table 4, analysis of FAME revealed different retention times, indicating the presence of different bioactive compounds. Of the several compounds, the 11 most important and predominant compounds were neochlorogenic acid, cyanidin-3-O-galactoside, chlorogenic acid, cyanidin-3-O-arabinoside, 5-carboxypyranocyanidin 3-O-β-glucopyranoside, quercetin-di-hexoside, quercetin-3-O-vicianoside, quercetin-3-O-robinobioside, quercetin-3-O-rutinoside, quercetin-3-O-galactoside, and quercetin-3-O-glucoside. 

### 3.9. Effects of FAME on LPS-Induced NO Production and Proinflammatory Cytokine Expression in RAW 264.7 Cells

**Figure 4:**
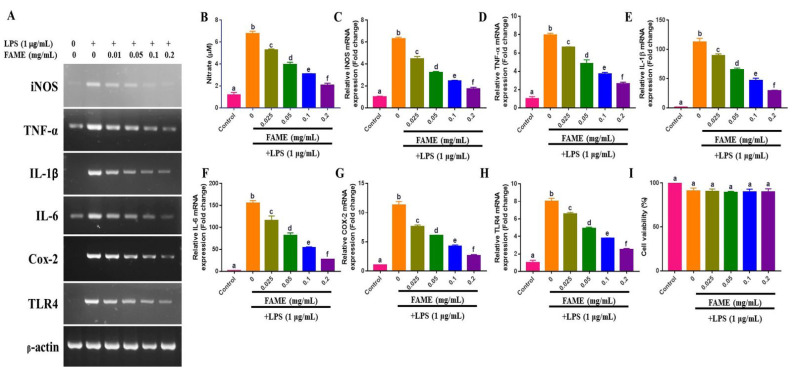
Production of NO and expression of different cytokines in LPS-treated RAW 264.7 cells upon treatment with FAME. Effects of FSE on LPS-induced NO production (**B**). Expression of proinflammatory cytokine mRNA determined by conventional PCR and qRT-PCR: iNOS (**A**,**C**), TNF-α (**A**,**D**), IL-1β (**A**,**E**), IL-6 (**A**,**F**), and Cox-2 (**A**,**G**). Expression of TLR4 mRNA (**A**,**H**) and cell viability (**I**). The data are presented as the mean ± SEM of three independent experiments. Different letters (a–f) above the bars indicate significant differences (*p* < 0.05) between groups.

To determine the potential anti-inflammatory effects of FAME, we measured the decrease in the production of NO and expression of proinflammatory cytokine mRNA. FAME significantly reduced LPS-induced NO production in RAW 264.7 cells in a concentration-dependent manner (0.025–0.2 mg/mL) (*p* < 0.05) (Figure 4B). Similarly, with an increase in the concentration of FAME, a corresponding decrease was noted in LPS-induced iNOS mRNA expression in RAW 264.7 cells. These results indicate a positive correlation between the decrease in NO production and the suppression of iNOS mRNA expression (Figure 4A,C). In addition, the tested concentration of FAME did not exert any cytotoxic effects on RAW 264.7 cells (Figure 4I). Moreover, FAME attenuated LPS-induced TNF-α, IL-1β, IL-6, Cox-2, and TLR4 mRNA expression in RAW 264.7 cells in a dose-dependent manner (0.025–0.2 mg/mL) (Figure 4A,D–H).

### 3.10. In Vivo Immunomodulatory Activities of FAME

#### 3.10.1. Acute Oral Toxicity Test of FAME

The acute oral toxicity test displayed no physical or clinical signs or mortality in the treated rats up to 14 days after the administration of FAME. In addition, no pathological lesions were observed in the organs of the treated rats. 

#### 3.10.2. Effect of FAME on Feed and Water Intake and on Body and Organ Weight 

There was no significant difference among the treatment groups in terms of feed and water consumption. In addition, the body weight changes were not significant. Furthermore, four organs (the spleen, liver, thymus, and both kidneys) were collected after the mice were sacrificed. Each organ was measured, and no significant difference was noted among the groups (data not shown).

#### 3.10.3. Effect of FAME on Neutrophil Migration and Phagocytosis

**Figure 5:**
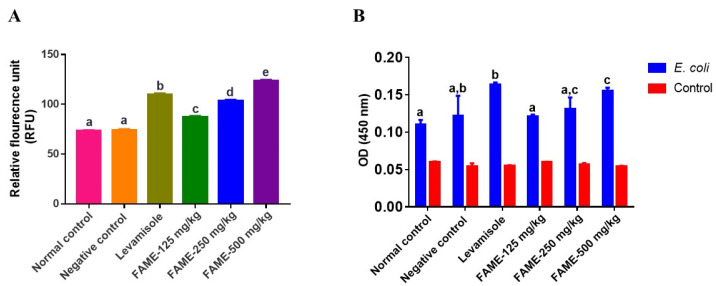
Effects of FAME on neutrophil migration (**A**) and phagocytic activity (**B**). Data are expressed as the mean ± SEM (*n* = 3). Different letters (a–e) above the bars indicate significant differences (*p* < 0.05) between groups.

In order to detect the primary immune function, the migration and phagocytosis activity of neutrophils were determined. Neutrophils are powerful effector cells that lead the first wave of host defense by destroying infectious agents through phagocytosis. They usually migrate quickly to sites of infection in order to counteract foreign invaders [44]. The migration of neutrophils was found to increase in a dose-dependent manner by 16.8%, 37.85%, and 65.51% in mice treated with 125, 250, and 500 mg/kg of FAME, respectively (Figure 5A) (*p* < 0.05). The phagocytic activity of neutrophils was assessed where *E. coli* was used as substrate. The phagocytic activity of neutrophils in mice treated with 250 and 500 mg/kg of FAME increased by 7.56% and 27.78%, respectively, showing a dose-dependent effect (Figure 5B). However, a significant difference (*p* < 0.05) was observed only for the highest dosage. 

#### 3.10.4. Effect of FAME on Splenocyte Proliferation

**Figure 6:**
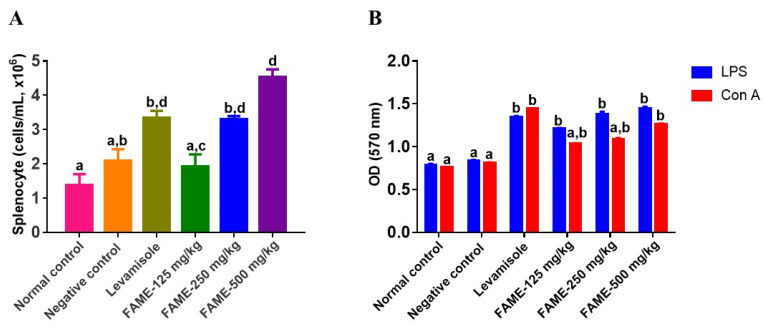
Effects of FAME on mouse splenocyte proliferation (**A**,**B**). (**A**) Total splenocyte count of different groups. (**B**) Ex vivo activation of splenocytes by LPS and Con A after 72 h of incubation. Data are expressed as the mean ± SEM (*n* = 3). Different letters (a–d) above the bars indicate significant differences (*p* < 0.05) between groups.

Cell-mediated immunity can be assessed by determining splenocyte proliferation. In general, splenocytes consist of different cell populations, such as macrophages and lymphocytes. Mice treated with FAME showed a significantly higher spleen cell count than the control mice, with the count increasing by 0.5-, 1.6-, and 4.7-fold at doses of 125, 250, and 500 mg/kg, respectively (Figure 6A). The effect of FAME on mouse splenocyte proliferation was studied using LPS (10 µg/mL) and Con A (5 µg/mL). LPS increased the proliferation by 44.9%, 65.03%, and 73%, whereas Con A increased the proliferation by 27.4%, 33.8%, and 54.9% at doses of 125, 250, and 500 mg/kg, respectively (Figure 6B). Thus, FAME increased splenocyte proliferation in a dose-dependent manner (*p* < 0.05). 

#### 3.10.5. Effect of FAME on T-Lymphocyte Differentiation

**Figure 7:**
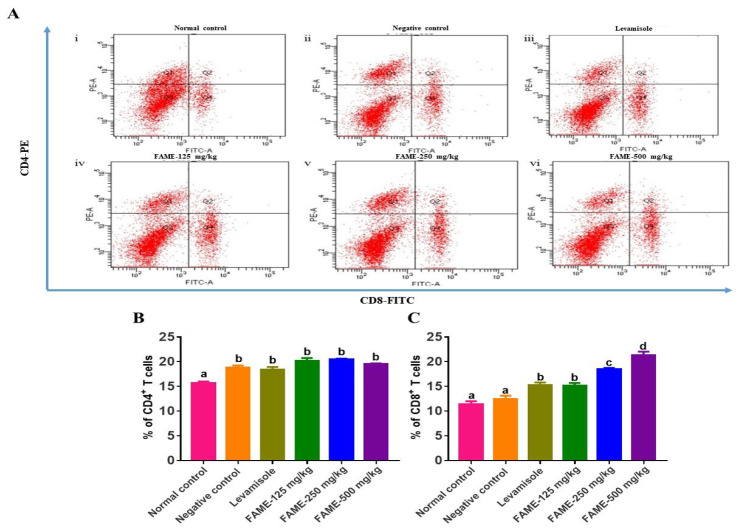
Flow cytometric immunophenotyping analysis of CD4^+^ (PE-conjugated) and CD8^+^ (FITC-conjugated) T-cell expression in splenocytes harvested from control and FAME-treated mice. (**A**) The percentages of CD4^+^ (region Q1) and CD8^+^ (region Q4) T cells in the spleen of mice. (**B**) The percentages of CD4^+^ cells and (**C**) the percentages of CD8^+^ cells. Data are expressed as the mean ± SEM (*n* = 3). Different letters (a–d) above the bars indicate significant differences (*p* < 0.05) between groups.

For T-lymphocyte phenotyping, flow cytometric analysis was performed. FAME-treated groups showed a dose-dependent increase in the expression of CD8^+^ T cells. Compared with the normal control mice, there was a significant increase in the expression of CD4^+^ T cells in the FAME-treated mice. At 125, 250, and 500 mg/kg, the expression levels of CD8^+^ cells increased by 15.13%, 18.46%, and 21.34%, respectively (Figure 7) (*p* < 0.05).

#### 3.10.6. Effect of FAME on Cytokine Expression

**Figure 8:**
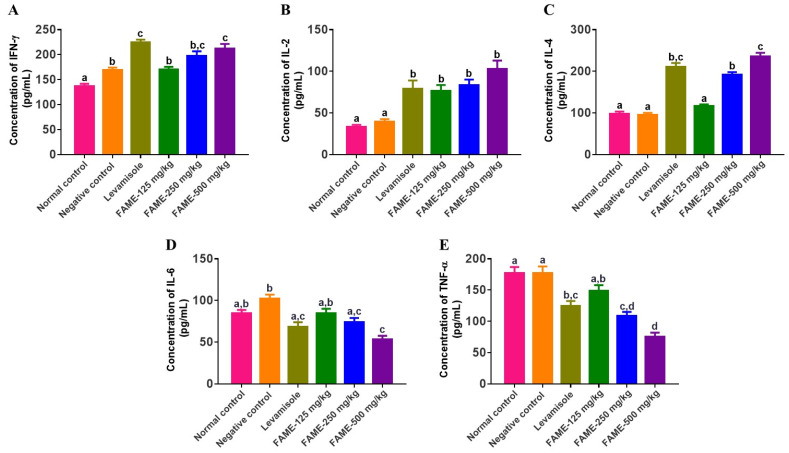
Cytokine profile of plasma obtained from mice treated with FAME (**A**–**E**). (**A**) IFN-γ, (**B**) IL-2, (**C**) IL-4, (**D**) IL-6, and (**E**) TNF-α. Data are expressed as the mean ± SEM (*n* = 6). Different letters (a–d) above the bars indicate significant differences (*p* < 0.05) between groups.

To investigate the role of FAME on Th1- and Th2-dependent cytokine expression, we determined the levels of cytokines in plasma samples obtained from different treatment groups by sandwich ELISA because cytokines play a central role in mediating the immunity, ranging from innate to cell-mediated immune response. Compared with negative control mice, IFN-γ levels increased by 22.51%, 41.35%, and 52.49% in mice treated with 125, 250, and 500 mg/kg of FAME, respectively. At the same time, IL-2 levels increased in a dose-dependent manner by 92.4%, 111.1%, and 158.9%, while IL-4 levels increased by 21.48%, 100.99%, and 146.35%, respectively (*p* < 0.05). However, FAME decreased the release of IL-6 by 17.24%, 27.39%, and 47.68% and that of TNF-α by 16.01%, 38.49%, and 57.59% at 125, 250, and 500 mg/kg, respectively (*p* < 0.05), as shown in Figure 8.

## 4. Discussion

The discovery of natural compounds from traditional medicinal plants has been explored since primitive times. Food supplement, pharmaceutical, and nutraceutical industries extensively use different herbal medicines, dietary supplements, nutrients, and remedies for treating various diseases [45]. The use of the fermentation process can reinforce the production of diverse bioactive secondary metabolites. The present results clearly revealed changes in the amounts of different compounds produced during fermentation processes; these could be interesting tools to produce and discover novel leads for treating various diseases, including immune disorders.

GABA is a nonprotein amino acid that has been widely documented as a functional component with health benefits [46]. As blackberries contain different powerful antioxidants and other functional constituents, different approaches, including fermentation, are being used to enhance specific health-beneficial compounds in these fruits and their derivatives. Because lactic acid bacteria are a fascinating group of microorganisms that are capable of yielding GABA, there is an opportunity to identify and isolate GABA-generating strains for use as starter cultures in the development and design of innovative functional fermented foods. GABA can be produced by the glutamic acid decarboxylase (GAD) pathway using *Lactobacillus* spp. by adding MSG as a precursor [10,47,48]. Therefore, we selected *L. plantarum* EJ2014 as the most appropriate GABA-producing lactic acid bacterium for our *A. melanocarpa* extract.

The production of GABA was significantly higher in FAME. In addition to acting as a nutritional component for *L. plantarum* EJ2014, the added yeast extract may have maintained bacterial growth, thereby facilitating the effective conversion of the MSG precursor into GABA. The addition of various concentrations of yeast extract has been reported to have a significant effect on the growth of *Lactobacillus* [49]. Similarly, the increase in acidity in the first 3 days of fermentation may be caused by the production of lactic acid by the growing *L. plantarum* EJ2014 strain. This decrease in pH may protect the extract from food pathogens. The subsequent increase in pH could be due to the decreased viable count of *L. plantarum*, which could produce less lactic acid after day 3 and accumulate the neutral amino acid GABA. This reduction in the viable cell count of *L. plantarum* EJ2014 may be caused by the accumulation of fermentation products and nutrient depletion with the increase in fermentation time. A similar trend was reported for *Lactobacilli* isolated from kimchi, in which fermentation caused a marked decrease in pH in an MSG-free MRS medium but resulted in an increase in pH in an MSG-containing medium [32]. In the present study, the reducing sugar contents were also measured during fermentation. The reducing sugar content in FAME decreased after 9 days of fermentation. *L. plantarum* EJ2014 is a homo-type strain that converts sugar into lactic acid, which in turn increases the acidity of fermented products [50]. 

In addition to the production and analysis of GABA, we determined and assessed other parameters of the extract during fermentation. The free radical scavenging activity of FAME was higher than that of the nonfermented extract. This may be due to the production of polyphenols, flavonoids, and anthocyanin in the extract, which could be triggered by fermentation. This is because the fermentation process causes the release of microbial enzymes, which in turn produce more freely available forms of plant phytochemicals, such as flavonoids, polyphenols, and anthocyanins [51]. Antioxidants play a significant role in immunomodulation [52]. These compounds regulate the immune system by interfering with gene expression, immune cell regulation, and cytokine synthesis [53,54]. The contents of different minerals significantly increased in FAME, in accordance with the findings of a previous study [55]. These micronutrients can regulate the overall disease by modulating immune functions and inflammation [56].

The production of GABA, different minerals, polyphenols, and flavonoids could have contributed to the outcome of the present study. Moreover, LC–MS/MS analysis of FAME confirmed the presence of 11 important bioactive isolates that could potentially be responsible for the immunomodulatory activities of FAME. However, as the production of GABA was also significant during the fermentation process, it can be said that GABA also greatly impacted the immunomodulation in mice. 

Macrophages play an important role in acute inflammation and in the persistence of proinflammatory cytokine expression, leading to chronic inflammatory conditions [57]. FAME significantly inhibits LPS-induced NO production and iNOS mRNA expression in RAW 264.7 cells. In addition, FAME consistently suppresses the expression of TNF-α, IL-1β, IL-6, and Cox-2 mRNA. Toll-like receptors recognize many pathogens and their pathogenic components (PAMPs) and initiate the innate immune response, thereby playing crucial roles in innate immunity [58,59]. Activated TLRs are vital for effective resistance against entering pathogens. LPS initially activates TLR4 to trigger the NF-κB signaling pathway, which regulates cytokine expression and the expression of many other inflammatory genes [60]. In the present study, PCR analysis revealed that FAME significantly inhibited TLR4 expression in a dose-dependent manner. Thus, FAME alleviates LPS-induced inflammation by inhibiting the expression of TLR4, indicating the potential suitability for the treatment of immune disorders.

Neutrophils and their migration are crucial for defending the body from invading agents and microorganisms [61]. They are recruited to the site of infection to engulf pathogens in response to inflammation. Hence, they are a vital part of the innate immune system [62]. FAME may influence the function of cells in the immune system in order to increase their migration and phagocytic activity. This increased neutrophil migration may be attributed to the enhanced GABA content in FAME. Moreover, the presence of anthocyanin may trigger the migration and invasion of neutrophils to the site of infection [63]. Previous research revealed that quercetin-3-O-rutinoside [64] and quercetin-3-O-robinobioside [65] have the potential to improve the innate immune response by accelerating neutrophil migration and phagocytosis activities. 

Splenocytes have the capability to boost immunity and hematopoietic functions [66]. In the present study, FAME-treated mice showed increased splenocyte proliferation. This increased splenocyte production can be indicative of enhanced humoral immunity, which can lead to increased immunoglobulin secretion by FAME. The high production of GABA may increase T-cell proliferation in experimental rat and mouse models [67,68] as GABA is essential for modulating T-cell proliferation. In addition, anthocyanin can aid splenocyte proliferation and thereby boost immunity [65].

FAME-treated mice showed higher T-lymphocyte differentiation than control mice. The regulatory effective immune function is significantly improved by the increased expression of CD4^+^ and CD8^+^ T cells because these two cell types constitute the majority of T lymphocytes and thus play pivotal roles in activating the innate immune response [69]. The enhanced expression of CD4^+^ and CD8^+^ T cells may be due to the presence of GABA [67,68]. GABA transporters have been reported to increase T-cell proliferation. Furthermore, flavonoids have been proven to be involved in the expression of CD4^+^ and CD8^+^ T cells [70]. The increase in CD8^+^ T cells may be caused by an increase in the level of IL-2, which is an important cytokine for the expansion of CD8^+^ T cells. 

FAME-treated mice showed an increase in the expression of IFN-γ, IL-2, and IL-4. IL-2 is mainly secreted by T cells, and it is important for T-cell proliferation, survival, and differentiation into effector cells [71]. The increasing level of CD8^+^ may contribute to the elevation of IFN-γ. This has also been manifested by the proliferation of T cells and the expression of CD4^+^ and CD8^+^ cells [71,72]. In addition, IL-2 is important for the generation of memory cells and for stimulating the secretion of IFN-γ [72]. The increase in IL-2 levels could also be caused by the increase in IFN-γ secretion. The predominance of T cells was manifested by increased IFN-γ and IL-4 production [73]. The dose-dependent reduction in the secretion of proinflammatory cytokines (TNF-α and IL-6) indicates the anti-inflammatory activity of FAME, which agrees with previous findings [74]. This may be due to the combined anti-inflammatory activity of both aronia extract and GABA in the fermented extract. GABA has been reported to decrease cytokine production [67]. Viking aronia extract has been found to inhibit LPS-stimulated IL-6 expression in murine splenocytes in a dose-dependent manner [74]. The GABA signaling system is clearly active in immune cells and can affect various cell functions, such as cytokine secretion, cell proliferation, phagocytic activity, and chemotaxis [68]. Hence, GABA plays a significant role in modulating the immune system [68]. Moreover, as a result of fermentation, aronia extract produces different flavonoids. Flavonoids have been shown to have potential cytokine modulation activities [75]. Therefore, FAME may exhibit potential immunomodulatory activities due to the combined effects of these compounds.

## 5. Conclusions

The present study revealed that aronia extract fermented by *L. plantarum* EJ2014 and fortified with a high GABA and phenolic compound content could increase the migration and phagocytic activities of neutrophils. In addition, the extract increased the number and proliferation of mouse splenocytes. It also increased the expression of CD4^+^ and CD8^+^ cells and of the cytokines IFN-γ, IL-2, and IL-4. However, it decreased the expression of TNF-α and IL-6, indicating its anti-inflammatory activity. These findings emphasize the potential of FAME as a promising functional food to ameliorate impaired immunity.

## Figures and Tables

**Table 1:** Treatment group description.

Groups	Description
Group I	Normal control group: received only water
Group II	Negative control group: received water and SRBC treatment
Group III	Positive control group: received a known immunostimulator (levamisole, 10 mg/kg)
Group IV	Treatment group: received 125 mg/kg of FAME
Group V	Treatment group: received 250 mg/kg of FAME
Group VI	Treatment group: received 500 mg/kg of FAME

**Table 2:** GABA production and changes in DPPH and other components of aronia extract fermented by *L. plantarum* EJ2014.

Targets	Unit	Before (0 Day) Fermentation	After (9 Days) Fermentation
Glutamic acid	μg/mL	13251 ± 9.44 ^a^	2014 ± 5.51 ^b^
GABA	μg/mL	N.D. ^a^	10412 ± 7.42 ^b^
DPPH	mg/mL	2.18 ± 0.48 ^a^	1.09 ± 0.28 ^b^
Potassium	mg/mL	9872.01 ^a^	10142.23 ^b^
Calcium	mg/mL	1442.05 ^a^	2014.09 ^b^
Magnesium	mg/mL	962.14 ^a^	1123.12 ^b^
Sodium	mg/mL	159.36 ^a^	282.14 ^b^
Iron	mg/mL	23.01 ^a^	25.54 ^b^
Phosphorus	mg/mL	16.26 ^a^	30.12 ^b^

Values represent the mean ± SD (*n* = 3). Significant differences (*p* < 0.05) indicated by different letters (a and b) were observed before and after fermentation. N.D.: not detected.

**Table 3:** Physicochemical properties of and polyphenol, flavonoid, and anthocyanin contents in aronia extract fermented by *L. plantarum* EJ2014.

FermentationTime (Days)	pH	Acidity (%, *w/v*)	Reducing Sugar Content (%)	Polyphenol Content (μg/mL)	Flavonoid Content (μg/mL)	Viable Cell Count (CFU/mL × 10^6^)	Anthocyanin Content (mg/100 mL)
0	4.41 ± 0.01 ^a^	0.74 ± 0.02 ^e^	4.1 ± 0.03 ^a^	213.79 ± 3.69 ^f^	34.23 ± 1.95 ^d^	4.86 ± 6.2 ^e^	0.0802 ^f^
1	3.86 ± 0.01 ^c^	0.84 ± 0.01 ^c d^	1.74 ± 0.02 ^b^	226.26 ± 3.81 ^e^	35.22 ± 2.65 ^d^	1250 ± 61 ^a^	0.0814 ^e^
3	3.31 ± 0.02 ^f^	1.08 ± 0.04 ^a^	1.62 ± 0.04 ^c^	262.12 ± 4.01 ^d^	36.22 ± 2.14 ^c^	938 ± 31 ^b^	0.0866 ^d^
5	3.62 ± 0.01 ^c^	0.82 ± 0.02 ^d^	1.44 ± 0.02 ^d^	280.25 ± 3.21 ^c^	37.08 ± 1.54 ^b^	155 ± 4.2 ^c^	0.0882 ^c^
7	3.84 ± 0.01 ^d^	0.93 ± 0.02 ^b^	1.02 ± 0.03 ^e^	291.24 ± 2.22 ^b^	37.61 ± 1.41 ^a^	78 ± 2.6 ^d^	0.0902 ^b^
9	4.01 ± 0.01 ^b^	0.86 ± 0.01 ^c^	0.86 ± 0.02 ^f^	312.54 ± 3.54 ^a^	38.12 ± 1.66 ^a^	42 ± 3.6 ^d e^	0.0912 ^a^

Values represent the mean ± SD of three independent experiments. Different letters for different fermentation times indicate significant difference (*p* < 0.05).

**Table 4:** The major bioactive compounds in FAME identified by LC–MS/MS analysis.

RT (min)	Formula	Δ ppm	Compounds	Activity
2.90	C_16_H_18_O_9_	0.990	Neochlorogenic acid	Antioxidant [33]
4.12	C_21_H_21_O_11_	1.541	Cyanidin-3-O-galactoside	Antioxidant and anti-inflammatory [34]
4.26	C_16_H_18_O_9_	2.032	Chlorogenic acid	Anti-inflmmatory, antioxidant, and antibacterial [35]
4.50	C_20_H_19_O_10_	0.780	Cyanidin-3-O-arabinoside	Antioxidant [36]
4.77	C_24_H_21_O_13_	0.857	5-Carboxypyranocyanidin 3-O-β-glucopyranoside	Antioxidant and anti-inflammatory [37]
5.71	C_27_H_31_O_17_	1.585	Quercetin-di-hexoside	Antioxidant and anti-inflammatory [38]
6.19	C_26_H_28_O_16_	0.551	Quercetin-3-O-vicianoside	Antioxidant [39]
6.46	C_27_H_30_O_16_	0.522	Quercetin-3-O-robinobioside	Antibacterial [40]
6.57	C_27_H_30_O_16_	0.522	Quercetin-3-O-rutinoside	Antioxidant [41]
6.67	C_21_H_20_O_12_	1.349	Quercetin-3-O-galactoside	Antimicrobial and antioxidant [42]
6.80	C_21_H_20_O_12_	1.349	Quercetin-3-O-glucoside	Antimicrobial [43]

## Data Availability

All data generated for this study are contained within the article and supplementary material.

## Supplementary Materials

The following is available online at https://www.mdpi.com/article/10.3390/antiox10081276/s1, Table S1: The LOD and LOQ of minerals, and accuracy of mineral contents for SRM 1869.
