# Supplementary material for: Aronia melanocarpa Extract Fermented by Lactobacillus plantarum EJ2014 Modulates Immune Response in Mice
Source: Antioxidants (Basel). 2021 Aug 11;10(8):1276. doi: 10.3390/antiox10081276 (PMC8389331; doi:10.3390/antiox10081276)
Supplement: Supplementary file 1 [file antioxidants-10-01276-s001.zip › antioxidants-1323942-supplementary.pdf]

Table S1. The LOD and LOQ of minerals, and accuracy of mineral contents for SRM 1869

Targets	LOD (μg/100g)	LOQ (μg/100g)	Recovery (%)	RSD (%)
Potassium	1.43	4.75	99.9	0.6
Calcium	2.63	8.77	101.8	1.4
Magnesium	3.36	1.12	99.6	0.4
Sodium	2.84	9.47	99.1	1.4
Iron	0.30	1.00	101.0	0.8
Phosphorus	1.01	3.35	101.2	0.1

The values are mean±S.D of replicates
Relative standard deviation (RSD)
Reference value indicates the mineral contents of SRM 1869 provided by National Institute of Standard and Technology (NIST) as certificate value
